# Editorial of the Special Issue: Signaling Molecules and Signal Transduction in Cells

**DOI:** 10.3390/ijms140611438

**Published:** 2013-05-29

**Authors:** Jens Schlossmann

**Affiliations:** Pharmacology and Toxicology, Institute of Pharmacy, University Regensburg, Universitätsstr, 31, D-93040 Regensburg, Germany; E-Mail: jens.schlossmann@chemie.uni-regensburg.de; Tel.: +49-941-943-4770; Fax: +49-941-943-4771

**Keywords:** signaling, signaling molecules, receptors, second messenger, kinases, phosphatases, posttranslational modifications, intercellular signaling

## Abstract

In the special issue “Signaling Molecules and Signal Transduction in Cells” authors were invited to submit papers regarding important and novel aspects of extra- and intracellular signaling which have implications on physiological and pathophysiological processes. These aspects included compounds which are involved in these processes, elucidation of signaling pathways, as well as novel techniques for the analysis of signaling pathways. In response, various novel and important topics are elucidated in this special issue.

## 1. Communication

Several of the manuscripts presented discuss compounds which might be involved in cellular apoptosis and thereby influence cancer or embryonic development.

The compound *Ginsenoside Rh2* (G-Rh2), derived from the plant Ginseng, acts anti-proliferative and pro-apoptotic. Its intracellular effects through apoptotic pathways were analyzed by Guo *et al. Rapamycin* is an inhibitor of mTOR (mammalian target of rapamycin) and thereby acts antiproliferative on some tumors [1]. Dai *et al.* studied the anti-tumor effect of rapamycin inducing apoptosis and autophagy on pancreatic cancer cells [2]. Sun *et al.* proffer that *JRS-15*, a derivative of xylocydine which was a novel cyclin-dependent kinase inhibitor, induced mitochondrial apoptosis in several cancer cell lines [3]. Kalimuthu *et al.* reviewed the effect of various *bioactive compounds from marine organisms* including sponges, actinomycetes and soft corals on the diverse apoptotic pathways of cancer cells [4].

The common mycotoxin *ochratoxin A* (OTA) is nephrotoxic, hepatotoxic and immunotoxic. The cytotoxic effects of OTA on mouse embryonic development inducing reactive oxygen species and mitochondrial apoptosis were studied by Hsuuw *et al.* [5]. Wnt morphogens are involved in various stages of development, including migration, cell polarity, proliferation and differentiation. Solis *et al.* reviewed the role of reggie/flotillin proteins for *Wnt* secretion and gradient formation and its effect on development [6]. Furthermore, Senarath-Yapa *et al.* reported the osteogenic potential of diverse signaling pathways including *Wnt*, *BMP*, *FGF and TGFβ* [7]. Adams *et al.* analyzed the toxic effect of cesium in plants. High concentrations of cesium inhibited plant growth inducing the jasmonate pathway and thereby probably modified potassium uptake machineries [8]. The differential role of defense response pathways, the *unfolded protein response (UPR)* and the *steroid response element* (*SREBP*) was studied by Bedoya-Perez *et al.* in the mosquito *Aedes aegypti* using the Cry11Aa toxin [9]. Further, the *UPR signaling pathways* in mammalian and their implications were reviewed by Carrara *et al.* [10].

G-Protein coupled receptors (GPCR) represent the most abundant class of mammalian membrane-bound receptors and are valuable pharmacological targets. The review by De Kejzer *et al.* described *prostaglandin E2 GPCR signaling* in dendritic cells in respect to the cellular life cycle [11]. *Resolvin* (resolution-phase interaction products) is a member of a novel family of aspirin-triggered short-lived autacoids synthesized during inflammation. Keinan *et al.* presented resolvin signaling pathways which could be used in oral health treatment [12].

Growth factors are important mediators of developmental processes. Mutations in the tyrosine kinase growth factor receptors are known to induce severe diseases, including the susceptibility to cancer. Therefore, the regulation of growth factor receptor signaling is essential for the understanding of physiology and pathophysiology of these proteins. In this regard, the link of *EGFR to the intracellular dynein IC2* was described by Pullikuth *et al.* [13]. Furthermore, mechanisms for the *spatial regulation of EGFR signaling including endocytosis* were elucidated by Ceresa *et al.* [14]. The mechanism of EGFR phosphorylation and its link to interacting proteins in uterine myoma was analyzed by Weissenbacher *et al.* [15]. They found that *EGFR Y845* phosphorylation probably interacted with Mucin-1 and cleaved Galectin-3 which could serve as a diagnostic tool for differentiation of benign and malign tumors. Regulation of endocytosis and cell signaling is an emerging role of *intersectins* which were summarized by Hunter *et al.* [16]. There were implications of intersectins in human diseases including Down syndrome, Alzheimer disease and cancer.

Muha *et al.* explained the role of *fibroblast growth factor (FGF)* and the *FGF receptors Heartless(Htl) and Breathless (Btl)* for development and differentiation in *Drosophila* [17]. Conidi *et al.* described that interference of peptide apatamers with growth factors e.g., TGFβ or EGFR could be suitable for the analysis of their signaling pathways in high throughput screening studies [18]. *Formyl peptide receptor 2 agonists*, their distinct signaling pathways and their involvement in immunological responses and cancer were reviewed by Cattaneo *et al.* [19].

Erythropoietin (EPO) induces erythropoiesis and is used as a pharmacological drug, e.g., as biosimilars for long-term treatment of anemia. However, EPO also acts on other types of cells, e.g., endothelial mediating proliferative and angiogenic effects and might be important for the therapeutic outcome. Trincavelli *et al.* discussed the effect of *EPO*, its derivatives and the *serine/threonine kinase receptor EPO-R* in endothelial cells, regarding desensitization/resensitization/expression using an *in vitro* model [20]. Hänel *et al.* discussed the role of *cytokines* in healthy and inflammatory skin diseases [21].

Functions of the *retinoid nuclear orphan receptor RORα* were reviewed by Du *et al.* implicating its role as tumor suppressor [22].

Mutations in the gene ATP2C cause the Hailey-Hailey skin disease in humans. ATP2C1 encodes *the secretory pathway calcium (Ca^2+^)-ATPase pump* (SPCA1). Micaroni *et al.* hypothesized that the gene ASTE1 influences ATP2C1 gene expression. ASTE1 dysregulation might induce cell death and tumor transformation [23].

*Nitric oxide* is an important signaling molecule which exerts pleiotropic functions. Its regulatory function in skeletal muscle during exercise was summarized by Suhr *et al.* [24]. Soluble guanylyl cyclases are activated by nitric oxide and thereby synthesize the second messenger cGMP. The detection of cGMP *in vivo* is an emerging field which was presented in comparison to cAMP by Sprenger *et al.* in a featured review paper [25].

Kinase cascades are essential for the intracellular signal transduction. In this regard, the *MAP kinase scaffold* was reviewed by Meister *et al.* [26] coordinating the cellular response. Candelori *et al.* presented a study regarding the gene *En-MAPK1* which was activated during pheromone signaling of the polar ciliate *Euplotes nobili* [27]. Furthermore, Joshi *et al.* described the regulation of T-Cell activation and function by *diacylglycerol kinases* [28].

Phosphorylation is controlled by protein phosphatases. Recently, atypical protein phosphatases were discovered which were structurally different from the known families of Ser/Thr- and Tyr-phosphatases. Sadatomi *et al.* set its focus on the *atypical phosphatases eyes absent* (EYA) which acted as dual Thr/Tyr-phosphatase and members of the *phosphoglycerate mutase* (PGAM) family (Sts-1, Sts-2, PGAM5) which exerted His-based Tyr-phosphatase activity [29].

The ubiquitination of proteins is a proteasomal degradation motif. However, ubiquitination is also used as an intracellular receptor signaling motif. In this regard, the *ubiquitination of Notch* and its signaling intracellular function was reviewed by Moretti *et al.* Small GTP-binding proteins are important regulators of intracellular signaling [30]. As an example, the function of protein *RhoA* in the intestinal epithelial barrier was summarized by Tong *et al.* [31]. A further posttranslational modification includes *SUMOylation*, e.g., on ATF3. Its role inhibiting prostate cancer cells was presented by Wang *et al.* [32].

The controlled release of compounds from cells is important for intercellular signaling and communication. Beyond various exocytosis mechanisms, the analysis and implication of *exosomes* for (patho)physiological processes is a topic which was reviewed in detail by Corrado *et al.* [33]. The composition of exosomes is not fully elucidated and is variable in various differentiated cells. However, there are numerous processes described involving exosomes with implications for health and disease, e.g., for immune response, neuronal signaling, rheumatoid arthritis and various cancers. The role of prostaglandins for intercellular neuronal signaling of endothelia, astrocytes and neurons and their involvement in neuronal injury was summarized by Takemiya *et al.* [34].

## 2. Conclusions

In summary, several important and novel aspects of intracellular and intercellular signaling in health and disease were highlighted in this special issue. However, signal transduction in and from cells is a huge field which can only partly be touched on in one special issue. Therefore, a further special issue covering more aspects of this engrossing field will follow in 2014.
